# The effects of using cognitive behavioural therapy to improve sleep for patients with delusions and hallucinations (the BEST study): study protocol for a randomized controlled trial

**DOI:** 10.1186/1745-6215-14-214

**Published:** 2013-07-11

**Authors:** Daniel Freeman, Helen Startup, Elissa Myers, Allison Harvey, John Geddes, Ly-Mee Yu, Zenobia Zaiwalla, Ramon Luengo-Fernandez, Russell Foster, Rachel Lister

**Affiliations:** 1 Department of Psychiatry, University of Oxford, Warneford Hospital, Oxford OX3 7JX, UK; 2 Department of Psychology, University of California, Berkeley, 3210 Tolman Hall, Berkeley CA 94720-1650, USA; 3 Centre for Statistics in Medicine, University of Oxford, Wolfson College Annexe, Linton Road, Oxford OX2 6UD, UK; 4 Oxford Non-respiratory Sleep Disorder Service, Oxford University Hospitals NHS Trust, John Radcliffe Hospital, Oxford OX3 9DU, UK; 5 Health Economics Research Centre, Department of Public Health, University of Oxford, Old Road Campus, Headington, Oxford OX3 7LF, UK; 6 Nuffield Laboratory of Ophthalmology, Nuffield Department of Clinical Neurosciences, University of Oxford, John Radcliffe Hospital, Oxford OX3 9DU, UK

**Keywords:** CBT, Delusions, Hallucinations, Insomnia, Schizophrenia, Sleep

## Abstract

**Background:**

Patients with psychosis frequently report difficulties getting or staying asleep (insomnia). Dissatisfaction with sleep is high. Insomnia should be treated in this group, but typically it is not even assessed. Importantly, recent evidence indicates that insomnia triggers and exacerbates delusions and hallucinations. The clinical implication is that if the insomnia is treated then the psychotic symptoms will significantly lessen. In a case series with 15 patients with persecutory delusions resistant to previous treatment this is exactly what we found: cognitive behavioural therapy for insomnia (CBT-I) led to large reductions in both the insomnia and delusions. The clear next step is a pilot randomized controlled test. The clinical aim is to test whether CBT-I can reduce both insomnia and psychotic symptoms. The trial will inform decisions for a definitive large-scale evaluation.

**Methods/design:**

We will carry out a randomized controlled trial (the Better Sleep Trial, or the BEST study) with 60 patients with distressing delusions or hallucinations in the context of a schizophrenia spectrum diagnosis. Half of the participants will be randomized to receive CBT-I, in addition to their standard treatment, for up to eight sessions over 12 weeks. The other half will continue with treatment as usual. Blind assessments will take place at 0 weeks, 12 weeks (post-treatment) and 24 weeks (follow-up). The primary outcome hypotheses are that CBT-I added to treatment as usual will improve sleep, delusions and hallucinations compared with only treatment as usual. All main analyses will be carried out at the end of the last follow-up assessments and will be based on the intention-to-treat principle. The trial is funded by the NHS National Institute for Health Research (NIHR) Research for Patient Benefit Programme. Data collection will be complete by the end of 2014.

**Discussion:**

This will be the first controlled test of CBT-I for patients with delusions and hallucinations. It will provide significant evidence for an easily administered intervention that is likely to prove very popular with patients experiencing the difficult-to-treat problems of delusions and hallucinations.

**Trial registration:**

Current Controlled Trials ISRCTN 33695128

## Background

Improvements in the treatment of delusions and hallucinations are greatly needed. One method for improving treatment involves drawing on developments in understanding these psychotic symptoms by targeting key putative causal factors one at a time (for example, [1-4]). Insomnia has been strongly implicated in the occurrence of psychotic symptoms but is yet to be a treatment target. Harvey et al. [5] explain: ‘Sleep disturbance is increasingly recognized as an important, but understudied, mechanism in the complex and multifactorial causation of the symptoms and functional disability associated with psychiatric disorders.’ Four recent studies by our group have established in cross-sectional and longitudinal designs that insomnia and paranoid delusions are linked [6-9]. For instance, in our initial study we found in a group of patients with persecutory delusions that 27% had severe clinical insomnia, 27% had clinical insomnia of moderate severity and 30% had subthreshold insomnia, that is, only 16% of patients were sleeping well. While it has long been known that sleep deprivation can bring on hallucinations [10] and that difficulties sleeping are one of the most common prodromal signs of psychotic disorders [11]. Moreover, Yang and Winkleman [12] have argued that their ‘data demonstrate that drug-free patients with chronic undifferentiated type schizophrenia suffer from profound disturbances in sleep continuity and sleep architecture.’ At a neurobiological level it has been suggested that the overactivity of dopamine D2 receptors in the striatum thought to underlie the positive symptoms of schizophrenia also enhances wakefulness [13]. The implication is that treating insomnia in patients with schizophrenia will lessen psychotic symptoms.

### Preliminary study

A large body of research, outside of psychosis groups, has found that cognitive behavioural interventions (CBT-I) are highly effective in treating insomnia [14,15]. In a recent case series, we used CBT-I with patients with psychosis for the first time [16]. Fifteen patients with persistent persecutory delusions and insomnia in the context of a psychotic disorder were individually given a standard-format, four-session CBT-I intervention. Outcome assessments were conducted at pre-treatment, post-treatment and one-month follow-up. Following the intervention, highly statistically significant reductions were found in levels of insomnia and the persecutory delusions. The effect sizes were large, and the changes were maintained at the time of the follow-up assessment. Nine patients reduced insomnia scores by at least 50% and 14 patients reduced insomnia scores by at least 25%. Five patients reduced persecutory thinking scores by at least 50% and eight patients reduced persecutory thinking by at least 25%. There were also significant reductions in levels of hallucinations, anxiety and depression. A more rigorous evaluation is required, which will test our trial procedures, and include an examination of the effects of the intervention on delusions and hallucinations.

### Pilot randomized controlled trial

A randomized controlled trial of CBT-I for patients with psychosis is now warranted. This pilot trial will: establish recruitment and follow-up rates; indicate levels of compliance with the treatment; determine whether actigraphy and sleep diaries can be used in this population; provide stronger evidence for the efficacy of the intervention (establishing treatment effect sizes); and indicate for whom the intervention is most suitable. It will test our procedures within a large mental-health unit in an NHS Trust hospital for recruitment from inpatient and outpatient services from different catchment areas. Therefore, there are three specific main aims: to establish that the trial procedures work (and fine tune where necessary), so that a Phase III trial can follow; to show that CBT-I is beneficial in improving sleep for individuals with distressing delusions or hallucinations; and to show that CBT-I has the added benefit of reducing delusions and hallucinations. The latter aims will establish treatment effect sizes. The primary outcome hypotheses are:

1. CBT-I added to treatment as usual will improve sleep in patients with psychosis compared with only treatment as usual.

2. CBT-I added to treatment as usual will reduce delusions and hallucinations compared with only treatment as usual.

The secondary hypotheses are:

1. Improvements in sleep and psychotic symptoms will be maintained over at least three months.

2. Improvement in sleep will be associated with improvements in psychotic symptoms.

3. CBT-I will lead to improvements in patient well-being and feelings of fatigue.

4. There will be indications that CBT-I is potentially cost-effective, by improving quality of life, reducing time in hospital over six months, and potentially generating NHS savings.

## Methods

### Research design

**Figure 1:**
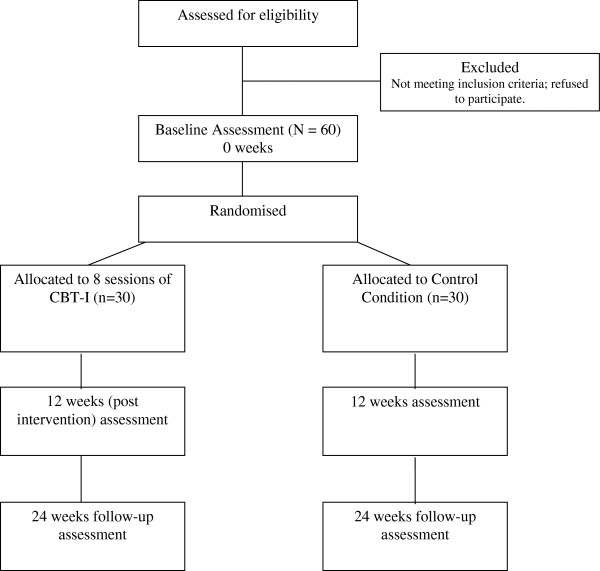
Flow diagram for the BEST study.

This is a prospective randomized pilot study to evaluate CBT-I in addition to standard psychiatric care versus standard psychiatric care alone in patients with distressing delusions or hallucinations (see Figure 1). A psychological intervention control group is not included in the design. We will instead examine how the treatment works by including repeated measures of insomnia and associated processes. Non-specific therapist factors will also be assessed [17]. A two-group design makes the successful completion of the trial much more feasible, while the addition of a third group would not add value in this instance. Randomization will be carried out independently by an on-line system developed by the Oxford Cognitive Health and Neuroscience Clinical Trials Unit. The randomizer programme will balance the following three variables: (i) sex (male, female), (ii) sleep problem severity (low, 15 to 21 on the Insomnia Severity Index (ISI); high, 22 to 28 on the ISI), and (iii) symptoms (hallucination only, delusions only, hallucinations and delusions). The trial therapist will inform patients of the randomization outcome, so that the research worker does not become unblinded. Rater assessments will be blind. All patients will be informed of allocation by the trial therapist to prevent the research assessors becoming unblinded. Precautionary strategies will include encouraging the therapist to consider room use and diary arrangements in the light of potential breaks of masking and reminding patients by the assessor not to talk about treatment allocation. Also, after the initial assessment, the assessor will not look at the patient’s clinical notes until the last of the ratings has been collected. The success of the blinding will be monitored; if there are breaks in the blinding, another assessor will be used from our research group. The reliability of the rater on the key interviewer measures will be formally assessed. The trial has received approval from the NHS Research Ethics Committee South Central, Oxford C (reference 12/SC/0138).

### Participants

Patients will be recruited from services across the Oxford Health NHS Foundation Trust, which covers a population of 1.1 million. We will seek referrals from all relevant outpatient and inpatient clinical teams who see patients with schizophrenia and related diagnoses. The inclusion criteria are: a current delusion or hallucination that has persisted for at least three months; a score of at least 2 on the distress scale of the Psychotic Symptom Rating Scales (PSYRATS) for either a delusion or hallucination [18]; a clinical diagnosis of schizophrenia, schizoaffective disorder or delusional disorder (that is, diagnosis of nonaffective psychosis (F2) in the International Classification of Diseases and Diagnostic and Statistical Manual IV); sleep difficulties lasting one month or longer with an ISI score of 15 or above (that is, above subthreshold insomnia). Participants must be aged between 18 and 65, and, where changes in medication are being made, entry to the study would not occur until at least a month after stabilization of dosage. It should be noted that we will be seeing patients when the main treatment, neuroleptic medication, has generally been tried at length and symptoms are relatively stable (persistent). It is increasingly recognized that the action of neuroleptic medication occurs rapidly [19,20]. Criteria for exclusion are: a primary diagnosis of sleep apnoea, alcohol or substance dependency; an organic syndrome or learning disability; a command of spoken English inadequate for engaging in therapy; and current individual CBT (though previous CBT experience is not an exclusion criterion). Written informed consent will be obtained from all participants.

### Planned interventions

The insomnia intervention will be provided in up to eight sessions over 12 weeks. The 12-week window will allow some flexibility for appointment times (it is quite common for patients in this group to fail to attend appointments) and the extension of intervals between the final two sessions. The exact number of sessions will depend on clinical appropriateness in the 12 week window. The main techniques, which are standard sleep interventions for CBT, are taken from four main sources [15,21-23]. The intervention is written in a manual, which we will develop further. Initially, the sessions focus upon psycho-education about sleep difficulties, assessment of the triggering and maintenance of sleep difficulties, and goal-setting. There is a checklist of factors likely to cause sleep difficulties. Based on the assessment, the active therapeutic techniques that are used included sleep hygiene, stimulus control therapy (for example, setting appropriate and regular sleep times, not doing anything else in the bed or bedroom apart from sleeping, not staying in bed if not able to sleep for longer than 20 to 30 minutes, stopping daytime naps), relaxation, and, less often, cognitive techniques to address unhelpful beliefs and attitudes about sleep, attentional bias, monitoring, and safety behaviours. The intervention is deliberately simplified, with the principal therapeutic technique being stimulus control; that is, learning to associate bed with sleep. Participants are also given written information as part of the intervention in the form of leaflets to read between sessions and on completion of the intervention. The intervention will be carried out by a qualified clinical psychologist, who carried out the case series therapy. Sessions will be taped for assessment of adherence and for competence [24]; when the purpose is clearly explained, we have found that 80% of patients with psychosis agree to recordings of sessions. We will obtain independent judgements on the quality of therapy using a random selection of therapy tapes. Patients will also be asked to assess the therapist’s empathy [25]. Standard care is delivered according to national and local service protocols and guidelines. Service use will be measured using the Client Service Receipt Inventory [26].

### Outcome measures

Before entry into the trial, patients will be assessed using the Duke Structured Interview Schedule for Sleep Disorder Diagnoses (Edinger JD, Kirby AC, Lineberger MD, Loiselle MM, Wohlgemuth WK, Means MK, unpublished). The key outcome measures will be levels of insomnia as assessed by the ISI [27], and levels of delusions and hallucinations as assessed by the PSYRATS [18,28]. These measures were used in our case series test. The ISI has been recommended by sleep experts as a key outcome measure for trials [29], whereas the PSYRATS is the best dimensional measure of psychotic symptoms and has been used in large clinical trials [30]. Both these key outcome measures have established psychometric properties and our case series patients found them easily understandable and helpful to assess relevant experiences.

Secondary outcome assessments will include patient-reported outcome measures: a psychometrically validated user-led assessment (CHOICE [31]) reflecting the priorities, such as self-confidence, peace of mind and a sense of being in control, of patients with psychosis; a measure of quality of life, which includes a simple assessment of subjective general health state (EQ-5D-5 levels [32]); and a self-reported measure of well-being (the Warwick-Edinburgh Mental Well-being Scale [33]). We will also include a self-reported measure of fatigue (Multidimensional Fatigue Inventory [34]), a second self-reported measure of sleep used in many insomnia trials (the Pittsburgh Sleep Quality Index [35]), a self-reported scale of suspicious thoughts (the Green et al. Paranoid Thoughts Scale [36]), and a standard psychiatric interviewer-rated assessment (the Positive and Negative Symptom Scale [37]). We will also ask participants to complete a brief sleep diary before each assessment point, and take part in actigraph assessment (an actigraph is a watch-like device that records movement or activity and ambient light to produce an objective assessment of sleep level); this will be an important opportunity to pilot these sleep assessments with this group. We will also record service use, including medication consumption (type, dose, and time taken), use of alcohol, illicit drugs and nicotine, physical health history, adverse events, and hospital admission data (including use of the Client Service Receipt Inventory [26]). We will also, if participants are willing, collect urine for 48 hrs before each assessment, so that levels of melatonin can be tested. For an exploratory examination of mediation we will include: the Beck Anxiety Inventory [38], the Beck Depression Inventory [39], a night-time worry scale and an activity diary. To test for moderation, we will also take a mouth swab to enable DNA analysis. We will also carry out a qualitative evaluation of the patients’ experiences and preferences concerning the CBT for insomnia intervention. This will be carried out for ten of the patients using interpretative phenomenological analysis. A semi-structured interview lasting 20–30 minutes will be used, developed in consultation with our service user advisors. Interviews will be tape recorded and then transcribed before analysis.

### Assessment and follow-up

The outcome measures will be completed before randomization (0 weeks), at the end of therapy (12 weeks) and at a follow-up (24 weeks). Paper copies will be kept of all assessments and data entered into an electronic database within one day of the assessment. All the data entry for the two main outcomes will be double checked. The baseline assessment must be completed before randomization. The end of therapy assessment must be carried out after therapy has been completed. We will endeavour to have the repeat assessments carried out at exactly the timings specified, but will allow a two-week window for the post-therapy assessment and a one-month window for the follow-up assessment. As discussed, all assessments will be carried out blind to group allocation, and if a break of blind does occur then another assessor will be brought in to re-establish blindness. Participants will be paid £15 for each assessment session to compensate them for their time.

### Assessment of safety

There were no adverse events in our initial case series evaluation of CBT-I. The following will be considered as adverse events: (i) all deaths; (ii) suicide attempts; (iii) serious violent incidents; (iv) admissions to secure units; and (v) formal complaints about therapy. We will also scrutinize any instances of patients being admitted to psychiatric hospital in the period of the therapy. These adverse events are likely to come to the attention of the assessor or therapist but we will also check medical notes at the end of each participant’s time in the trial. Responses to adverse events will be determined on a case-by-case basis by the trial team.

### Statistical analysis

All main analyses will be carried out at the end of the last follow-up assessments (that is, there will be no interim analyses) and will be based on the intention-to-treat principle, with due consideration given to potential biases arising from loss to follow-up. Parameter estimates and confidence intervals of continuous outcomes will be obtained using analysis of covariance, adjusting for baseline variables. (We will also look at the effect of treatment difference by controlling for initial overall symptom severity (assessed by the Positive and Negative Symptom Scale) and medication use as a sensitivity analysis). Absolute risk difference and relative risk will be calculated for binary outcomes. Rates of attrition and loss to follow-up will be calculated for each time point. We will also assess the correlations of each measure across all time points. These findings will be used to inform the sample size for the definitive trial. For the health economic analysis, quality adjusted life-years will be used as an outcome measure, by combining EQ-5D quality-of-life information with survival data. A cost-utility analysis will be performed, in which the differences in mean costs between the CBT and standard care groups will be divided by the difference in mean outcomes between the two groups. The sample size of 60 is based on our collective recruitment experience from previous and current studies, and is considered adequate for obtaining reasonably reliable sample size estimates [40]. The patients will be recruited over 15 months. A fully detailed statistical analysis plan will be written and signed off by the investigator prior to any analysis by the trial statistician.

## Discussion

The importance of sleep in the occurrence of psychiatric problems is becoming increasingly recognized [5,41]. The BEST study will be the first randomized controlled test of cognitive behaviour therapy for insomnia in patients with nonaffective psychosis. The trial is funded for 24 months and staff began in post in October 2012. Final outcome assessments will be complete by the end of August 2014. Therefore, the study results will become available in 2015. We predict that the intervention will not only improve sleep but lessen distressing positive symptoms of psychosis. Our experience is that the intervention is popular since it focuses on a problem that the patient recognizes and hence there is a clear shared outcome goal. In the study we will be able to compare self-reported and more objective markers of sleep (for example, actigraphy, melatonin), while we will assess delusions and hallucinations multidimensionally (for example, frequency, preoccupation, distress, and interference). If the study is successful, we anticipate that the next step will be a definitive Phase III clinical trial. We think it likely that in the future the treatment of sleep problems will be an important tool in the reduction, and perhaps prevention, of distressing psychotic experiences.

### Trial status

Patients began to enter the trial in December 2012. Recruitment will continue for 15 months.

## Abbreviations

CBT-I: Cognitive behavioural therapy for insomnia; ISI: Insomnia Severity Index; NIHR: National Institute for Health Research; PSYRATS: Psychotic Symptom Rating Scales.

## Competing interests

The authors declare that they have no competing interests.

## Authors’ contributions

DF took the main responsibility for drafting the study protocol. DF is the principal investigator. L-MY has the main responsibility for the trial outcome analyses. RL-F has the main responsibility for the health economic analysis. EM is the trial therapist and RL is the research assistant. DF, HS, and AH provide the training and supervision for the trial therapist. All authors contributed to the design of the trial and read and approved the final manuscript.
